# Evaluation of specific and non-specific immune response of four vaccines for caseous lymphadenitis in sheep challenged

**DOI:** 10.14202/vetworld.2018.1272-1276

**Published:** 2018-09-17

**Authors:** Sohier M. Syame, Azza S. M. Abuelnaga, Eman S. Ibrahim, Ashraf S. Hakim

**Affiliations:** Department of Microbiology and Immunology, National Research Centre, Dokki, Cairo, Egypt

**Keywords:** *Corynebacterium pseudotuberculosis*, immune response, lymphocyte, phospholipase D, vaccine

## Abstract

**Background:**

Caseous lymphadenitis (CLA) is a serious disease affects sheep and goat, caused by *Corynebacterium pseudotuberculosis*. Due to it is non-treatable disease, so the effective preventive vaccines are considered a significant way to combat the disease. All strains of *C. pseudotuberculosis* have several virulence factors that associated with their cell invasion, survival, and proliferation such as phospholipase D (PLD), outer lipid coat, and secreted proteases.

**Aim:**

The present study was directed to perform a comparative innate and acquired immune response assessment of different four vaccine formulas to evoke protection against induced (CLA) challenge in sheep.

**Materials and Methods:**

Negative ELISA (free of CLA) 15 local breed male (Balady) sheep were divided into five groups, each has received a different vaccine while the control has received saline buffer. The first vaccine composed of toxoid PLD alone the second composed of toxoid PLD with bacterin (formalinkilled bacteria), the third vaccine composed of toxoid PLD plus covaccine 8, while the fourth one composed of toxoid PLD plus locally produced polyvalent clostridial vaccine. The specific immune response was evaluated through lymphocyte proliferation assay using ELISA BrdU kit, while the non-specific response was estimated by superoxide anion production and lysozyme activity assays.

**Results:**

The study revealed that PLD toxoid could evoke the highest specific immune response, showing a stimulation index (9.12%). On the other hand, combined toxoid ↱PLD with bacterin followed by PLD toxoid showed a significant increase in the non-specific innate immune response.

**Conclusion:**

The present study indicated that the toxoid PLD alone vaccine was most efficient and provided innate and acquired immune response in animals against CLA.

## Introduction

Caseous lymphadenitis (CLA) is a chronic bacterial infectious disease of sheep and goats, caused by inhalation or ingestion of the Gram-positive bacterium *Corynebacterium pseudotuberculosis*, and is responsible for many economic losses [1,2]. Controlling CLA with antibiotics is unuseful since the bacteria have surrounded by a thick capsule, which can protect it inside the abscesses [3]. The disease presents in two different ways, the external, also known as superficial or cutaneous form which is characterized by the development of abscesses within the subcutaneous tissue or superficial lymph nodes. Pepin *et al*. [4] reported that at the first 24 h post-infection of lambs, microabscesses appear in the cortical region of the lymph node draining the site of inoculation with increasing of different cellular infiltration. Later, small nodules of mineralization formed the classic “onion ring” sectioned presentation and regarded as virtually pathognomonic for CLA [5]. The second form of CLA is a visceral form which characterized by the formation of lesions in the host body, commonly the internal lymph nodes (primarily the mediastinal lymph nodes) or lungs also the kidneys, liver, or the mammary glands and less frequently infected the brain, heart, spinal cord, uterus, testes, and joints [6]. The pathogenesis of *C. pseudotuberculosis* is attributed to its virulence factors, the major one is an exotoxin called phospholipase D (PLD) that increasing vascular permeability and enhances dissemination of the bacteria by damaging endothelial cells. Another virulence factor is an outer lipid somatic coat that protects the bacteria from hydrolytic enzymes in host phagocytes where the bacteria replicate and release when rupture [7,8].

As *C. pseudotuberculosis* can replicate within phagocytic cells, as a facultative intracellular pathogen, cellular immunity is believed to be necessary for efficient and effective protection. Not only lymphocytes are essential player in the specific immune response against the bacterium, but also macrophages have an essential role in the development of cellular either innate through secretion of bactericidal molecules and primary lysosomes or recognized acquired immunity [9].

In our investigation, we assessed the efficacy of cell-mediated innate and acquired immune response for different four vaccine formulas to trigger protection against CLA in sheep.

## Materials and Methods

### Ethical approval

The study was performed following the animal experimentation ethics.

### Animals and experimental design

A total of 15 sheep, approximately 8-10 months old, were pretested as negative ELISA (free of CLA) and divided into five groups each group constituted three animals. Four groups were vaccinated by four different vaccine formulas, and the fifth group was kept as a non-vaccinated control group.

### Groups and vaccine formula

#### Group A (vaccine 1): Toxoid PLD

Preparation of culture filtrate from isolated *C. pseudotuberculosis* biovar 1 (sheep origin), according to Brown *et al*. [10] with our modification as described by Syame [11] and Selim *et al*. [12]. 1 ml of the filtrate was mixed with 1 ml of oil adjuvant vaccine, as each formed dose (2 ml) contained 23 μg PLD.

#### Group B (vaccine 2): Toxoid - bacterin

It is composed of formalin-killed *C. pseudotuberculosis* whole cells [13] mixed with the toxoid PLD vaccine as 164 killed bacteria cells and 23 μg PLD/1 ml.

#### Group C (vaccine 3): Toxoid PLD with covaccine 8

Covaccine 8 is an imported vaccine formulated from a mixture of clostridial toxins, obtained from Schering plough animal health, the mixture contained 23 μg PLD in 40 ml of covaccine 8 [14].

#### Group D (vaccine 4)

Toxoid PLD vaccine combined with the polyvalent clostridial vaccine (*Clostridium perfringens*, *C. tetani*, *C. septicum*, *C. chauvoei*, and *C. novyi*) as 40 ml of polyvalent clostridial vaccine mixed with 6 g lyophilized powder of formulated culture filtrate of PLD [11]. The polyvalent is a local vaccine prepared by Veterinary Serum and Vaccine Research Institute, Abbasia.

#### Group E (control)

Non vaccinated animals.

### Vaccination and experimental challenge

Groups 1, 2, 3, and 4 were vaccinated with the previously mentioned vaccine formula by S/C inoculation of 2 ml dose of vaccine in the middle third of the neck. All the vaccinated animals were revaccinated after 3 weeks of the first vaccination. After 3 weeks of the last vaccination, all five groups including the control non-vaccinated sheep were challenged with virulent biovar 1 sheep origin isolate with 2 ml suspension containing 4×10^6^ CFU intradermally as 1 ml in both sides of the neck. The non-specific cellular immune response represented by stimulated macrophages was measured at 2 weeks after the challenge while the specific excited lymphocytes response was assessed at 4 weeks.

### Collection of antiserum

Blood samples were obtained from the jugular vein and drawn through a syringe without anticoagulant 2 weeks after the challenge, kept in slanting position for about 2 h, and after that centrifuged at 1600× g for 25 min at 4°C. The supernatant was collected in sterile vials, and the serum was kept at 57°C in a water bath for 30 min to inactivate the complement system, then stored at −20°C for testing.

#### Superoxide anion production assay

The assay was performed in triplicate at a sterile tissue culture plate; a working solution was prepared (cytochrome-C purchased from Sigma 200 mg/1 ml Hanks balanced salt solution (HBSS) and prewarmed to 37°C. Each well received a mixture of 100 µl of working solution and 50 µl of the sera with different vaccine formula in HBSS; the control wells received the working solution only. The superoxide dismutase (4 units/well) was added to the mixture and served as a blank reference well. The plate was incubated at 37°C in a humidified 5% CO_2_ tension for 2 h, then read at 570 nm using ELISA reader [15]. More superoxide anion production, more reduction of cytochrome C red color and was calculated by linear regression analysis. Purified superoxide dismutase was used to confirm the specificity of superoxide production.

#### Lysozyme activity assay

Tested sera (100 µl) were added to 2 ml of a suspension of *Micrococcus lysodeikticus* ATCC 4698 Sigma (0.2 mg/ml) in a 0.05 M sodium phosphate buffer (pH 6.2). The reactions were carried out at a 20°C, and optical density at 530 nm was measured between 5 min and 20 min on a spectrophotometer. A lysozyme activity unit was defined as the amount of enzyme producing a decrease in optical density of 0.001/min against standard curves [16,17].

### Preparation of peripheral blood mononuclear cells (PBMCs)

Five mm of the jugular vein blood sample were obtained from each animal through heparinized Vacutainer (10 IU/ml). Heparinized blood was diluted 1:1 with sterile phosphate buffered saline (PBS) and overlaid on the Ficoll separation medium by 3:1 in 20 ml test tubes, then centrifuged the tubes at 800 rpm for 10 min at 4°C. The PBMCs↱ in the interface layer represented both lymphocytes and monocytes were harvested and immediately washed 2 times with sterile PBS, then centrifuged at 1500 rpm for 10 min at room temperature. After washing, PBMCs were resuspended in 2 ml RPMI 1640 medium [18].

#### Evaluation of specific cellular immune response by the lymphocyte proliferation assay (LPA) [19].

LPA measures the ability of lymphocyte to undergo a clonal proliferation when stimulated by foreign antigen. The amount of proliferation is detected using cell proliferation ELISA BrdU (Bromodeoxyuridine) colorimetric kit (Roche) as a thymidine analog incorporated into DNA replication and can be detected immunocytochemically after partial denaturation of double-strand DNA by a specific anti-BrdU monoclonal antibody. 10 µl of the reconstituted cells were then mixed with 90 µl of 0.4% trypan blue stain and hemocytometer checked. The number of lymphocytes /ml of each sample was calculated:


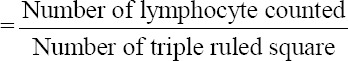


100 µl of 5×10^5^ viable lymphocytes cells μml were dispensed in tissue culture plate wells, the plate was pulsed with the kit reagents followed the instructions and the plate was incubated at room temperature until a sufficient color was developed. The absorbance of the samples was measured in ELISA reader at 370 nm.

### Statistical analysis

Statistical analysis was done using model GLM of SAS software version (6.12) [20].

The superoxide anion production was calculated as follows:

Blank well - test well × 15.87*

As 15.87 is a coefficient calculation based on the quantity of solution per well and dimension of the well.

The stimulation index (SI) was calculated as follows:


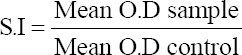


>1 indicates a positive proliferation response.

## Results and Discussion

The present investigation was directed to evaluate both the non-specific and specific cellular efficacy of toxoid PLD (toxoid of *C. pseudotuberculosis)* as a single vaccine compared to other used combined vaccine formula to evoke protection against *C. pseudotuberculosis* virulent strain locally isolated from sheep suffered from CLA in Egypt. Once the organism is intracellular, the antitoxins antibodies probably have a little impact on the recovery from infection so that the cellular dependent responses may be essential in eliminating the organism [21].

During the early stages of the disease, macrophage infiltration was directed to combat and the organism, by oxidative expressed in the production of bactericidal oxygen free radicals and non-oxidative mechanism (enhancement of lysozymes activity). Lysozyme is a mucolytic enzyme able to induce damage of peptidoglycan, so it is highly effective, especially against Gram-positive bacteria [22,23].

The achieved data in Table-1 exhibited a significant increase in the level of superoxide anion production of simulated macrophage vaccinated groups, compared with the control one. The highest increased in Group B (toxoid PLD+bacterin) little followed by Group A (Toxoid PLD alone), then Groups D and C nearly similar response. Furthermore, near results obtained in Table-2 showed that the significant lysozyme activity of simulated macrophage was the highest increased in Group B followed by Group A, then C and the least one was represented in Group D. These outcomes were consistent with those of other studies [24,25] and may be attributed to the presence of somatic proteins associated with bacterin which induce the production of IFN-γ, a very important cytokine related to innate immune response and macrophage stimulation [26].

**Table-1:** Effect of the four vaccine formula on superoxide anion liberation from triggered macrophages in tested sheep sera after 2 weeks from the challenge.

Type of vaccine	Mean OD	Mean OD	Superoxide anion (ng/ml)
Toxoid PLD (Group A)	0.192±0.02	Blank 0.420±0.04	3.61
Toxoid PLD+Bacterin (Group B)	0.186±0.01		3.71
Toxoid PLD+Covaccine 8 (Group C)	0.252±0.04		2.67
Toxoid PLD+polyvalent clostridial vaccine (Group D)	0.257±0.04		2.6
Control group (Group E)	0.308±0.06		1.77

Data are presented as mean±SE. P<0.05. SE=Standard error, PLD=Phospholipase D

**Table-2:** Effect of the four vaccine formula on lysozyme activity of stimulated macrophages in tested sheep sera after 2 weeks from the challenge.

Type of vaccine	Mean OD	Lysozyme activity
Group A	1.552±0.02	100 units
Group B	1.379±0.01	177 units
Group C	2.378±0.04	78 units
Group D	2.069±0.02	48 units
Group E	2.556±0.04	0 unit

Data are presented as mean±SE. p<0.05. SE=Standard error

From the other sides, the acquired immune response which may result after vaccination was evaluated by the LPA using ELISA BrdU colorimetric kit. The data in Table-3 showed a marked significant positive lymphocyte proliferation response in vaccinated groups, pointed to the highest (SI=9.12) represented by Group A (toxoid PLD) followed by other groups by far great extent. The results indicated that PLD stimulated the specific cellular immune response and highly suggested to confer the organism and this concern was agreed with other data [27,28].

**Table-3:** Mean OD value of lymphocyte proliferation response after 4 weeks from a challenge.

Type of vaccine	Mean OD	Lysozyme activity
Group A	0.575±0.03	9.12
Group B	0.305±0.02	4.8
Group C	0.361±0.02	5.73
Group D	0.290±0.01	4.6
Group E	0.063±0.002	1

Data are presented as mean±SE. p<0.05. SE=Standard error

## Conclusion

Drawing from the obtained data of this work, the most efficient protection against CLA was provided in animals vaccinated with toxoid PLD alone. The mentioned vaccine evoked the highest stimulation index, which expressed specific lymphocyte proliferation response by a big margin with the other used combined vaccines. In the same direction, it was able to induce the innate immune response as a macrophage stimulation mechanism in a long approximate border.

## Authors’ Contributions

SMS supervised the experiment. ASMA and ESI shared in experimental steps, vaccination, challenge and collection of samples. ASH conducted the immunological assays. SMS and ASH prepared and reviewed the manuscript. All authors read and approved the final manuscript.
